# Hypochondriasis

**Published:** 1887-08-13

**Authors:** 


### The Doctor's Pulpit.

### HYPOCHONDRIASIS.

There is a great advantage in giving a thing a name, especially a long one. When that is done we at least think we know something about it, which is perhaps the next happiest thing to really knowing. What is hypochondriasis? It is quite safe to defy any doctor to tell us with precision. It would be an amusing and, to a thinking mind, an instructive thing to ask twenty of the most learned Fellows of the College of Physicians each to give a definition of hypochondriasis. We venture to say that no two of their definitions would be identical, and perhaps hardly even similar. And yet it would be a mistake to suppose that there is no such thing in existence as is signified by the word. Hypochondriasis, it is true, is not a separate entity which can be weighed, measured, and accurately defined like a jelly-fish or a piece of cheese. It is the name, not of a thing, but for an assemblage of symptoms which in their completeness make up a condition of misery and melancholy well deserving of the longest and most incomprehensible word in the doctor's unapproachable vocabulary. We will not venture upon a definition. The man of definitions and logomachies is generally a poor-brained creature who defines because he is not able to do anything better or more original. Nearly every grown-up man knows what a hypochondriac is, and the best thing we can hope for those who are not grown up, is that they may never know.

There was a newspaper proprietor in Yorkshire, twenty years ago, who was only well about once in every three or four years. He was a dreadful Radical, and although he usually had legs as thick as mill-posts, and a liver which made him as miserable as even a Conservative could desire, he could always get both legs and liver right during a contested election. No sooner was his borough declared vacant than he began to rise early in the morning, tear about the streets from dawn till dark, make speeches, abuse opponents, encourage friends, and otherwise to find life a most interesting and enjoyable thing. He was the very type of a hypochondriac. What was the cause of his hypochondria? Pure laziness! If he had consulted a learned member of the faculty, as he probably often did, no doubt the man of physic would have found a thousand scientific and legitimate reasons why he should suffer as he did, and equally no doubt he would have suggested a thousand modern and scientific remedies. Everybody knows the famous cure suggested by—was it Abernethy? for this disease: "Live upon sixpence a day, and earn it." That is by no means fashionable advice, and would not nowadays take a man to Harley Street, or keep him there. Nevertheless it was then, and is now, worth a two-guinea fee. Not long ago a wealthy hotel-keeper got what he called a "fit of the blues," and a very prolonged fit it was. He consulted his family doctor, who had the honesty to tell him to wear a blue ribbon, and if necessary to give up his business—he was rich enough to do so. He did not take the advice, but "consulted a physician," as he expressed it, in Finsbury Square. The physician prescribed for him, and received his two guineas; but the patient had ultimately to go back to his family doctor again, who simply repeated his former advice. This time it was taken, and with the best results. There was a rather unusual termination to the episode: When the family doctor sent in his account the now retired publican disputed some of the items, and added, besides, that he had been "obliged to consult a physician." To this the doctor replied that the physician's prescription might or might not be worth the two guineas paid for it, but that his own counsel about the blue ribbon would not be paid for too highly if a thousand guineas were charged.

What is to be said about the cause and cure of this miserable and unworthy disease? The causes are mostly mental laziness, bodily inactivity, and errors in eating and drinking. By way of cure, poor old gentlemen are sometimes told to ride on horseback, and to follow religiously a prescribed routine of eating, drinking, and other wholesome habits: and very excellent advice it is when the patient is too old ever to be really changed in habit and character. But in the case of younger men it is absurd to turn them into such miserable valetudinarians. A physician of our acquaintance not infrequently says to a hypochondriac: "The best thing that could happen to you would be that you should be stolen or sold into slavery, and there compelled, by a moderately kind but stern master, to work hard in the open air all day long, and to live as well-treated slaves usually have to live. No doubt you would be wretched for a few months, but you would be quite cured in a year. Go now and do your best to live as you would have to live if you were somebody's slave." Not once in a hundred times does the patient value the advice. Both men and women, like children, love a "medicine made easy." It is probable that the cave-man and the river-drift man knew nothing of hypochondria; and it is laughable to suppose that the lion or tiger ever suffers from the disease, except when living a civilised and wealthy life in a menagerie or "zoo." What a reflection on the superior intelligence of man that the lion and the tiger should be altogether healthier and happier than he!

But what, after all, is the best cure for hypochondriasis? Six words will explain it: "Hard physical work with plain fare." As the Scripture says, "He that hath ears to hear, let him hear."

*(To be continued.)*

